# Medication-related problems among hospitalized pregnant women in a tertiary teaching hospital in Ethiopia: a prospective observational study

**DOI:** 10.1186/s12884-020-03433-6

**Published:** 2020-11-26

**Authors:** Seid Mussa Ahmed, Johanne Sundby, Yesuf Ahmed Aragaw, Hedvig Nordeng

**Affiliations:** 1 Department of Community Medicine and Global Health, Institute of Health and Society, Faculty of Medicine, University of Oslo, Oslo, Norway; 2 Division of Social and Administrative Pharmacy, School of Pharmacy, Faculty of Health Sciences, Jimma Institute of Health, Jimma University, Jimma, Ethiopia; 3 Department of Obstetrics and Gynaecology, Faculty of Medical Sciences, Jimma Institute of Health, Jimma University, Jimma, Ethiopia; 4 Pharmacoepidemiology and Drug Safety Research Group, Department of Pharmacy, Faculty of Mathematics and Natural Sciences, University of Oslo, Oslo, Norway; 5 Department of Child Health and Development, Norwegian Institute of Public Health, Oslo, Norway

**Keywords:** Medication-related problem, Maternity, Gynaecology, Pregnancy, Clinical significance, Iron supplementation

## Abstract

**Background:**

Studies on medication-related problems (MRPs) among pregnant women are scarce, despite the potential consequences for both mother and child. This study aimed to describe the prevalence, clinical significance, and risk factors for MRPs among hospitalized pregnant or postpartum women at Jimma University Medical Centre (JUMC) in Ethiopia.

**Methods:**

A prospective follow-up and clinical audit of 1117 hospitalized pregnant or postpartum women in the maternity and gynaecology wards at JUMC was carried out between February and June 2017. Patients were followed throughout their stay in the hospital to assess the presence and development of MRPs. Pre-tested data extraction form and an interview-guided structured questionnaire were used to collect data. Descriptive statistics were used to describe MRPs. Logistic regression analysis was used to identify factors associated with MRPs.

**Results:**

One or more MRPs occurred among 323 (28.9%) study participants, mostly in relation to lack of iron supplementation. A total of 278 (70.6%) of all MRPs were considered to be of moderate to high clinical significance. When excluding MRPs due to iron from the analysis, chronic disease (adjusted OR 1.91; 95% CI 1.02, 3.58), medication use prior to admission (adjusted OR 2.38; 95% CI 1.24, 4.56), nulliparity (adjusted OR 1.99; 95% CI 1.22, 3.24) and multiparity (adjusted OR 1.91; 95% CI 1.17, 3.12) were significantly associated with experiencing an MRP.

**Conclusions:**

Nearly 3 out of 10 hospitalized pregnant women at JUMC had one or more MRPs. The need for additional iron therapy was by far the most common type of MRP. Improved adherence to guidelines on iron supplementation are required. Multidisciplinary approaches including physicians, nurses, anesthesia professionals and clinical pharmacists in the maternity and gynaecology wards could possibly prevent MRPs and promote patient safety for women and children.

**Supplementary Information:**

The online version contains supplementary material available at 10.1186/s12884-020-03433-6.

## Background

A medication-related problem (MRP) is defined as an unwanted event or circumstance involving medication therapy that actually or potentially interferes with desired health outcomes [1, 2]. Studies have shown that the prevalence of MRPs among hospitalized pregnant patients varies from 42 to 83% [3, 4]. Patients who have MRPs are likely to have a longer hospital stay, recurrent hospital admissions, reduced quality of life, increased overall health care cost, and even an increased risk of morbidity and mortality [4–6].

Only a handful of studies have examined the frequency and nature of MRPs occurring in an obstetric hospital inpatient setting [3, 4]. A recently published Norwegian study of 212 pregnant women in an inpatient setting identified 105 MRPs occurring in 42% of pregnant women. “Need for additional drug” (46.7%), “adverse drug reaction” (20.0%), and “patient adherence” (10.5%) were the most common categories of MRPs. The most common medication groups involved in the MRPs were drugs acting on the respiratory system (25%; mainly nasal decongestants, 9%), anti-infectives for systemic use (18%; mainly antibiotics for systemic use, 8%), and drugs acting on blood and blood-forming organs (16%; mainly iron supplementation, 14%) [4]. A study from Australia identified 400 potential MRPs in 171 of 205 hospitalized pregnant women. The majority of MRPs were of low clinical significance (92%). The most common types of MRPs were “incomplete medications charted on admission” (28%), “dose too high” (26%), “incomplete drug order” (15%), and “additional medication required” (13%). The therapeutic groups most commonly associated with MRPs were medications for the alimentary tract and metabolism, mainly aperients (18%) and vitamins (13%), followed by drugs for the nervous system, mainly analgesics (25%) and antidepressants (4%) [3]. In addition to these two studies, a few studies have evaluated medication errors in obstetric and maternity wards [7–9].

In Ethiopia, previous studies have focused mainly on prescription drug use, drug use patterns, and self-medication practices among pregnant outpatients attending obstetrics and gynaecology departments [10–13]. Notably, none of these prior investigations involved the identification of MRPs and were conducted in ambulatory pregnant patients. Although few MRPs identification studies were performed among hospitalized patients in the country, all focused on the non-pregnant patient population [14–17]. Thus far, no study has investigated MRPs in an obstetrics group in a hospital setting in Ethiopia. Therefore, the objective of the present study was to determine the prevalence, clinical significance, and risk factors of MRPs occurring in hospitalized pregnant women in the maternity and gynaecology wards of Jimma University Medical Centre (JUMC) in Southwest Ethiopia.

## Methods

### Sample size

The required sample size for this study was calculated assuming a 50% proportion of MRPs, 5% level of precision, 3% error margin, and 5% possible non-response rate, making the minimum sample size 1121 pregnant women.

### Study setting

A facility-based prospective observational study was conducted in the maternity and gynaecology wards of JUMC, a tertiary level public teaching hospital located in Jimma City in southwest Ethiopia, 350 km from the capital city of Addis Ababa. It is the only teaching and tertiary level care hospital in southwest Ethiopia, with a catchment population of approximately 20 million people [18, 19]. Most of the pregnant women referred to the hospital come from rural areas, where many deliveries are attended at home [20, 21]. The Department of Obstetrics and Gynaecology at JUMC provides specialized health services for approximately 7580 inpatients and 11,590 outpatients each year, with a bed capacity of 265. The department has two wards (gynaecology and maternity/labour), one general gynaecological outpatient clinic, one antenatal care outpatient clinic, and one family planning clinic. Women are treated at the gynaecology inpatient ward before 28 weeks of pregnancy. Most pregnant women admitted to this ward have elective and/or spontaneous abortions, hyperemesis gravidarum (HEG), or other early pregnancy complications. After 28 weeks of pregnancy, women are admitted to the maternity/labour inpatient ward. Women having a vaginal delivery give birth in the labour ward and are transferred to the maternity ward after delivery. If the mother and baby are healthy, they are discharged at the earliest possible time after delivery, usually within 1–2 days. Women having a caesarean delivery are transferred to the maternity ward and usually stay for 72 h.

### Data collection and procedures

Women in the maternity and gynaecology wards at JUMC between February and June 2017 were invited to participate in the study during normal working hours. Patients were informed of the aim and procedures of the study, and written informed consent was obtained from each study participant. Women who were under 18 years of age, too ill to participate, who declined to participate, were hard of hearing, unable to speak or with mental illness, admitted for a brief time (< 4 h), and non-pregnant women admitted to the gynaecology ward were excluded from the study.

The women were followed throughout their stay in the hospital to assess the presence and development of MRPs. Pre-tested data extraction form and an interview-guided structured questionnaire were used to collect the data. Five trained clinical pharmacists (data abstraction and MRP assessment) and four trained nurses (the questionnaire) from JUMC collected the data.

Information on the reason for admission, diagnoses, dosage regimens, discharge medications, maternal and perinatal outcomes, laboratory results, and length of hospital stay was collected by reviewing patients’ medical cards and medication charts. The card and chart reviews were performed for each patient on the first day of admission and repeated on subsequent days. The questionnaire was used to collect maternal socio-demographic characteristics, obstetric history, past medical history and medication experience, social drug use, and medicinal plant use.

### MRP identification and assessment

MRPs were classified into eight categories: need for additional drug therapy, unnecessary drug therapy, dose too low, dose too high, ineffective drug, adverse drug reactions, noncompliance [1], and other, subdivided into need for additional laboratory test and/or incomplete drug order (Additional file 1) [3].

MRPs were identified by reviewing patients’ medical cards and medication charts, and patient interviews about medication use while in the hospital. A panel of experts comprised of senior clinical pharmacists and experienced obstetricians/gynaecologists identified MRPs and classified them into categories as recommended by Cipolle et al. [1]. The panel of experts further refined the MRP identification and classification method for the study setting in accordance with Ethiopian standard treatment guidelines and literature reviews (Additional file 1) [3, 22–25].

The clinical significance of each MRP was categorized as level 1 or level 2 [3]. Level 1 are those MRPs that have low potential to give rise to patient discomfort or clinical deterioration whereas level 2 are MRPs that have moderate to severe potential to give rise to patient discomfort or clinical deterioration [3, 26]. At first, experienced clinical pharmacists identifying MRPs in the wards assessed and classified the clinical significance, and subsequently discussed by the panel of experts. Differences in opinion on severity level of MRPs were discussed until consensus was reached. The description of the MRPs, their clinical significance, and the medication (s) involved were recorded using a purpose-built data collection tool.

The classification of medications involved in MRPs was performed per the World Health Organization (WHO) Anatomical Therapeutic Chemical Classification system (ATC) that categorizes medications into 14 main groups [27].

### Statistical analysis

Descriptive statistics were used to calculate percentages. The results were presented as medians and ranges. Univariate and multivariate logistic regression analyses were used to calculate odds ratios (ORs) with 95% confidence intervals (CIs) and identify risk factors associated with MRPs. The independent variables were patient-related factors (age, level of education, marital status, occupation, religion, ethnic group, family size, residence place, alcohol use status, and khat chewing), disease-related factors (patient admission ward, i.e., gynaecology or maternity ward; chronic disease; obstetrics category, i.e., caesarean or vaginal delivery; duration of hospital stay), pregnancy related factors (parity, gravidity, gestational age, adverse pregnancy outcome [current and previous], status of anaemia), medicine-related factors (medicines used during admission or prior to admission, ferrous sulphate supplementation, medicinal plant use, concomitant use of medicinal plants), facility-related factors (walking distance to the nearest health facility, and availability of preferred medication for a specific condition). Explanatory variables with *p* ≤ 0.05 in the univariate analysis were entered into a multivariate logistic regression model to determine independent risk factors of MRPs. As iron supplements were involved in 165 (41.9%) of the MRPs, a post hoc logistic regression analysis was performed excluding iron. All data were analysed using the Statistical Package for the Social Sciences (SPSS) software version 25.0 for Windows (IBM® SPSS® Statistics, Armonk).

## Results

### Study population characteristics

A total of 1137 pregnant and nursing women were asked to participate in the study, and 1121 (98.6%) accepted. Responses from four women were incomplete, leaving 1117 women in the final study population, 88.8% from the maternity ward (611 vaginal deliveries, 372 caesarean sections, and 9 did not proceed to parturition) and 11.2% from the gynaecology ward. The median patient age was 25 years (range 18–45 years). Most of the women were either primiparous or multiparous (40% each). Most women gave birth at term (65.8%) through vaginal labour (54.7%). Five percent of the women had one or more chronic diseases. The median length of hospital stay was 3 days, ranging from 5 h to 60 days, and most of the patients (59.7%) stayed ≤3 days in the hospital. A fifth of the women (19.4%) had adverse pregnancy outcomes in the current pregnancy, and 1 in 10 women (11.0%) had a history of adverse pregnancy outcomes. Detailed sociodemographic characteristics and clinical data are summarized in Tables 1 and 2. In this study, concomitant use of phytomedicines and conventional medicines was assessed by identifying women who used both during pregnancy for the same or different illnesses.

**Table 1:** Risk factors of medication-related problems ^a^

Characteristics	No. (%) 1117 (100)	MRPs		Crude OR (95% CI)	Adjusted OR (95% CI) ^b^
		No MRPs	≥1 MRP		
**Age (years)**					
≤ 20	223 (20.0)	169	54	1	1
21–25	388 (34.7)	278	110	1.24 [0.85, 1.81]	1.34 [0.91, 1.98]
26–30	320 (28.7)	216	104	**1.51 [1.03, 2.22]**	**1.63 [1.07, 2.50]**
≥ 31	186 (16.7)	131	55	1.31 [0.85, 2.04]	1.31 [0.79, 2.19]
**Residence place**					
Urban	595 (53.3)	423	172	1	
Rural	522 (46.7)	371	151	1.00 [0.77, 1.30]	
**Chronic disease**					
Yes	56 (5.0)	34	22	1	
No	1061 (95.0)	760	301	0.61 [0.35, 1.06]	
**Medicinal plant used in current pregnancy**					
Yes	319 (28.6%)	228	91	1	
No	798 (71.4%)	566	232	1.03 [0.77, 1.37]	
**Alcohol consumers**					
Yes	46 (4.1)	33	13	1	
No	1071 (95.9)	761	310	1.03 [0.54, 1.99]	
***Khat*** **chewers** ^**c**^					
Yes	65 (5.8)	44	21	1	
No	1052 (94.2)	750	302	0.84 [0.49, 1.44]	
**No. of medicines during admission**					
< 5 medication	631 (57.3)	455	176	1	
≥ 5 medication	470 (42.7)	329	141	1.11 [0.85, 1.44]	
**No. of medicines prior to admission**					
No past medication	165 (14.8)	119	46	1	
Only one past medication	666 (59.6)	466	200	1.11 [0.76, 1.62]	
Two or more past medications	286 (25.6)	209	77	0.95 [0.62, 1.46]	
**Duration of hospital stay**					
≤ 3 days	667 (59.7)	482	185	1	
> 3 days	450 (40.3)	312	138	1.15 [0.89, 1.50]	
**Gestational age**					
Preterm pregnancy	231 (20.7)	150	81	1	1
Term pregnancy	735 (65.8)	539	196	**0.67 [0.49, 0.92]**	0.79 [0.51, 1.23]
Post term pregnancy	62 (5.6)	46	16	0.64 [0.34, 1.21]	0.72 [0.36, 1.46]
Others	89 (8.0)	59	30	0.94 [0.56, 1.58]	1.04 [0.58, 1.89]
**Patient ward**					
Gynaecology ward	125 (11.2)	78	47	1	1
Maternity ward	992 (88.8)	716	276	**0.64 [0.43, 0.94]**	0.76 [0.44, 1.30]
**Adverse pregnancy outcome in the current pregnancy**					
Yes	217 (19.4)	149	68	1	
No or not yet delivered and outcome not yet known	900 (80.6)	645	255	0.87 [0.63, 1.20]	
**Previous adverse pregnancy outcome**					
Yes	123 (11.0)	84	39	1	
No/not Applicable	994 (89.0)	710	284	0.86 [0.58, 1.29]	
**Parity**					
Primiparous	227 (20.3)	181	46	1	1
Nulliparous	441 (39.5)	308	133	**1.70 [1.16, 2.49]**	**1.82 [1.23, 2.69]**
Multiparous	449 (40.2)	305	144	**1.86 [1.27, 2.72]**	**1.73 [1.16, 2.59]**

**Bold**, statistically significant, *P* < 0.05

^a^ Numbers may not add up to 100% due to missing values

^b^ Adjusted for age, gestational age, patient ward and parity

^c^ *Khat* (*Catha edulis*) plant leaves are chewed by people to attain a state of euphoria and stimulation

**Table 2:** Risk factors of medication-related problems, excluding iron preparations ^a^

Variable category	No. (%) 1117 (100)	Non-Iron MRPs ^b^		Crude OR (95% CI)	Adjusted OR (95% CI) ^c^
		No MRPs	≥1 MRP		
**Age**					
≤ 20	223 (20.0)	190	33	1	
21–25	388 (34.7)	325	63	1.12 [0.71, 1.76]	
26–30	320 (28.7)	259	61	1.36 [0.85, 2.16]	
≥ 31	186 (16.7)	159	27	0.98 [0.56, 1.70]	
**Residence place**					
Urban	595 (53.3)	493	102	1	
Rural	522 (46.7)	440	82	0.90 [0.66, 1.24]	
**Chronic disease**					
No	1061 (95.0)	892	169	1	1
Yes	56 (5.0)	41	15	**1.93 [1.05, 3.57]**	**1.91 [1.02, 3.58]**
**Medicinal plant used in current pregnancy**					
Yes	319 (28.6%)	265	54	1	
No	798 (71.4%)	668	130	0.96 [0.68, 1.35]	
**Alcohol consumers**					
Yes	46 (4.1)	37	9	1	
No	1071 (95.9)	896	175	0.80 [0.38, 1.69]	
***Khat*** **chewers** ^**d**^					
Yes	65 (5.8)	59	6	1	
No	1052 (94.2)	874	178	2.00 [0.85, 4.71]	
**No. of medicines during admission**					
< 5 medication	631 (57.3)	520	111	1	
≥ 5 medication	470 (42.7)	399	71	0.83 [0.60, 1.15]	
**No. of medicines prior to admission**					
No past medication	165 (14.8)	152	13	1	1
Only one past medication	666 (59.6)	546	120	**2.57 [1.41, 4.68]**	**2.38 [1.24, 4.56]**
Two or more past medications	286 (25.6)	235	51	**2.54 [1.34, 4.82]**	**2.21 [1.12, 4.38]**
**Duration of hospital stay**					
≤ 3 days	667 (59.7)	555	112	1	
> 3 days	450 (40.3)	378	72	0.94 [0.68, 1.31]	
**Gestational age**					
Preterm pregnancy	231 (20.7)	199	32	1	
Term pregnancy	735 (65.8)	608	127	1.30 [0.85, 1.98]	
Post term pregnancy	62 (5.6)	51	11	1.34 [0.63, 2.84]	
Others	89 (8.0)	75	14	1.16 [0.59, 2.30]	
**Patient ward**					
Gynaecology ward	125 (11.2)	113	12	1	1
Maternity ward	992 (88.8)	820	172	**1.98 [1.07, 3.66]**	1.34 [0.68, 3.58]
**Adverse pregnancy outcome in the current pregnancy**					
Yes	217 (19.4)	185	32	1	
No or not yet delivered and outcome not yet known	900 (80.6)	748	152	1.18 [0.78, 1.78]	
**Previous adverse pregnancy outcome**					
Yes	123 (11.0)	106	17	1	
No/not Applicable	994 (89.0)	827	167	1.26 [0.74, 2.16]	
**Parity**					
Primiparous	227 (20.3)	203	24	1	1
Nulliparous	441 (39.5)	360	81	**1.90 [1.17, 3.10]**	**1.99 [1.22, 3.24]**
Multiparous	449 (40.2)	370	79	**1.81 [1.11, 3.94]**	**1.91 [1.17, 3.12]**

**Bold**, statistically significant, *P* < 0.05

Abbreviations: OR: odd ratio; CI: Confidence interval

^a^ Numbers may not add up to 100% due to missing values

^b^ No MRPs = MRPs due to other medications + Patients with No MRPs. ≥1 MRP = ≥1 MRPs due to iron sulphate

^c^ Adjusted for chronic disease, number of medicines prior to admission, patient ward and parity

^d^ *Khat* (*Catha edulis*) plant leaves are chewed by people to attain a state of euphoria and stimulation

### Medicine use during pregnancy and admission

The majority of the women had used one or more medications during pregnancy (85.2%), whereas 28.6% of women (Tables 1 and 2) had used medicinal plants. Ferrous sulphate was the most commonly used medication prior to hospital admission (97.3%). Furthermore, 271 (24.3%) women concomitantly used medicinal plants and medicines prior to admission.

During admission, the median number of prescribed medications was 3 per patient (range: 0–24; Additional file 2) and 42.7% of the participants were taking ≥ 5 medications (Tables 1 and 2). The three most common types of medications given during the women’s hospital stay were pitocin (63.7%), normal saline (38.9%), and ceftriaxone (36.0%) (Additional file 3). Ferrous sulphate (54.4%), cephalexin (30.4%), and metronidazole (25.0%) were the three most common medications prescribed at hospital discharge (Additional file 4).

### Medication use-related problems

One or more MRPs occurred among 28.9% of the study participants: 23.7% had one MRP, 4.2% had two MRPs, 0.8% had three MRPs, and 0.2% had four MRPs. A total of 394 discrete MRPs were noted. The highest number of MRPs, 87.6%, was identified among women admitted in the maternity ward (228 MRPs among those with vaginal delivery, 114 MRPs among caesarean sections, and 3 among those not yet delivered; Table 1, Additional file 2).

Two hundred and seventy-eight (70.6%) of all MRPs were considered to be of moderate to high clinical significance and classified as level 2 MRPs. One hundred sixty five (41.9%) of the total MRPs (133 (47.8%) of level 2 and 32 (39.0%) of level 1 MRPs) were due to iron treatment/supplementation.

Chart reviews were usually performed twice, with a range of one to three reviews (Additional file 2).

In 14.5% of patients, lack of chart recording or documentation of medication administration occurred, most commonly for ceftriaxone, anaesthetic drugs, and intravenous fluids in relation to caesarean section in the surgical delivery room. The assessment panel of experts agreed not to consider this an MRP, as the medications were appropriately administered to the patients.

The types of MRPs according to the eight main MRP categories are presented in Table 3. The most common MRP types were: need for additional drug therapy (*n* = 236 cases, 73.1%), need for an additional laboratory test (*n* = 41 cases, 12.7%), unnecessary drug therapy (*n* = 38 cases, 11.8%), and too low dosage (*n* = 38 cases, 11.8%; Table 3).

**Table 3:** Overview of Medication Related Problems (MRP) according to frequency and types

MRPs Category	Type of MRP	n (%) ^a^	Example
Indication	Needs additional drug therapy	236 (73.1)	Patient is asthmatic, but is not getting the recommended drug i.e. salbutamol puff PRN
	Unnecessary drug therapy	38 (11.8)	Patient is on ceftriaxone 1 g IV BID but there is no indication of infection in the diagnosis
Effectivenes	Dosage too low	38 (11.8)	Cephalexin 500 mg once PO daily given to patient to treat infection, PO BID daily is recommended
	Ineffective drug product	12 (3.7)	HIV/AIDS (immunocompromised) and MRSA infected patient who was on wound care was on metronidazole and cephalexin treatment (less effective), instead patient was put on more effective drug, vancomycin 500 mg IV BID for 10 days
Safety	Dosage too high	12 (3.7)	Patient is on ceftriaxone 2 g IV bid to treat chorioamnionitis which is high dose, 1 g IV BID is enough
	Adverse drug reaction	2 (0.6)	Patient received furosemide and gentamicin concurrently. One increases toxicity of the other by pharmacodynamic synergism; alternative drug chlorothiazide was used in place of furosemide
Compliance	Non-compliance	12 (3.7)	Anti-D immunoglobulin is available in the hospital, but the patient couldn’t afford and was not injected
Other categories	Need for an additional laboratory test	41 (12.7)	Patient haematocrit value is not registered to recommend or not iron supplementation or treatment
	Incomplete drug order	3 (0.9)	Patient is prescribed with methyldopa 250 mg (mild pre-eclampsia), but duration was not indicated
Total MRPs		394 (100.0)	

Abbreviations: *BID* Bis in die (twice daily); *IV* Intravenous; mg, milligram; *MRP* medication-related problem; *MRSA* Methicillin-resistant *Staphylococcus aureus*; *SCAP* Severe Community-Acquired Pneumonia; PRN, as needed; *PO* Per os (by mouth or orally)

^a^ Percentage is calculated taking those with ≥ MRP as denominator, *N* = 323. Percentage may exceed 100% due to more than one MRP per patient

A more detailed overview of the specific causes of MRPs and medications involved in MRPs are found in Additional file 5 and Additional file 6, respectively.

**Fig. 1:**
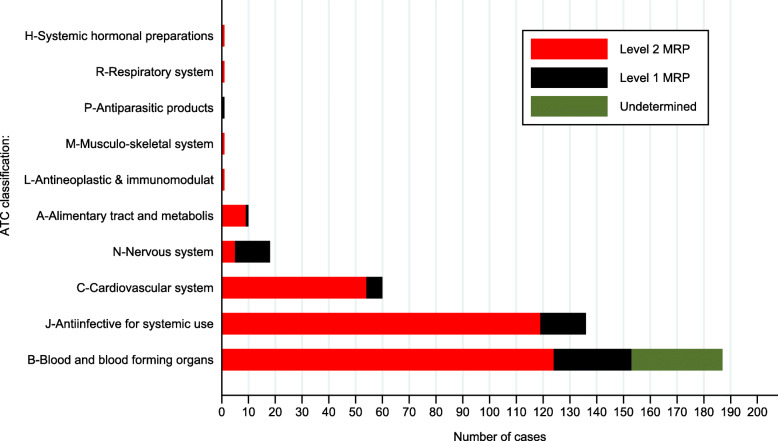
Overview of the medication groups (by ATC classification system) most commonly involved in MRPs according to severity of the MRP

As indicated in Fig. 1, the most common therapeutic group implicated in MRPs were medications acting on blood and blood-forming organs, mainly ferrous sulphate (35.3%), followed by anti-infectives for systemic use, largely cephalexin and metronidazole (each 9.4%).

### Factors contributing to MRPs

Nulliparous (adjusted OR 1.82; 95% CI 1.23, 2.69) and multiparous (adjusted OR 1.73; 95% CI 1.16, 2.59) women were significantly more likely to experience MRP than primiparous women. Similarly, women aged > 26 years (adjusted OR 1.63; 95% CI 1.07, 2.50) were more likely to experience MRPs than their counterparts (Table 1). However, in a post hoc analysis excluding MRPs due to iron from the analysis, only parity was maintained as a risk factor. Additional risk factors, including chronic disease (adjusted OR 1.91; 95% CI 1.02, 3.58) and past medication use (adjusted OR 2.38; 95% CI 1.24, 4.56; adjusted OR 2.21; 95% CI 1.12, 4.38) were associated with a significantly increased likelihood of experiencing an MRP than their counterparts (Table 2).

All MRPs were considered clinically significant for the patients. Most commonly, “need for additional medication therapy” included untreated disease conditions, mainly anaemia or an absence of anti-infection prophylaxis, as in patients at risk of infection due to retained placenta not receiving prophylactic antibiotic. Additional file 5 describes in detail the different types and causes of MRPs.

## Discussion

This study provides new knowledge about the prevalence, clinical significance, risk factors, and medications implicated in MRPs among hospitalized pregnant women in a resource-limited setting. To the best of our knowledge, this is the first study in Ethiopia to investigate the extent of MRPs in hospitalized pregnant women. Approximately 3 out of 10 pregnant women had one or more MRPs, mostly in relation to a lack of iron supplementation. More than 7 out of 10 MRPs were considered to be of moderate to severe clinical relevance. This high magnitude and the frequency of these MRPs suggests problems inherent in the day-to-day practices of the study wards. These problems can potentially be improved through internal audits, improved routines, and systemic changes, including multidisciplinary collaboration, training, and increased staff.

In the present study, nearly one-third of pregnant and nursing women encountered at least one MRP. This is lower than reported in studies of Norwegian [4] and Australian [3] pregnant and lactating inpatients, as 42.0 and 83.4% of the study participants, respectively, experienced at least one MRP. This large variation in prevalence is likely a reflection of the methodological differences between the studies, especially the process of medication reconciliation and medication chart reviews. Another possible reason for the difference in results could be differences in health care systems, the study populations, and disease distribution.

In agreement with the study conducted in Norway [4], 6 out of 10 MRPs in our study concerned the need for additional medication therapy because of untreated illness, mostly in relation to anaemia. In contrast, the study from Australia found that “incomplete medications charted on admission” (28%) and “incomplete drug order” (15%) were the two most prevalent MRP categories [3]. The difference may be due to the fact that the Australian study group was able to perform a formal medication reconciliation, which we were not able to do.

Medications acting on the blood and blood-forming organs, anti-infectives for systemic use, cardiovascular drugs, and drugs acting on the nervous system were the medications most commonly involved in MRPs in our study. These findings are relatively similar to prior studies. Antibiotics and iron were the second and third most frequently associated medications in MRPs in the previous Norwegian study [4]. In Australia, most MRPs were related to the alimentary tract and metabolism, and drugs for the nervous system (largely analgesics and antidepressants) [3]. These medication groups may need to be specific focus of the global perspective of MRPs.

Identifying patients with an increased risk of MRPs can be a useful guide for prioritizing tasks in the ward. Our study implies that the focus should be on women with a chronic disease and on women with prior medication use, which is in line with the findings of previous studies [3, 4]. Interestingly, parity was a risk factor for both iron-related MRPs and non-iron-related MRPs, whereas chronic disease and prior medication use were only risk factors for non-iron-related MRPs. This may be due to patients with chronic diseases being more likely to use multiple medications, increasing the risk of drug interactions and non-adherence, which in turn increases the risk of MRPs.

Almost half of the moderate to severe (level 2) MRPs (47.8%) were due to lack of iron supplementation. Another 34 cases had unknown haematocrit due to forgetfulness/lack of time to order standard blood tests or to record patient haematocrit values. Maternal iron deficiency anaemia during pregnancy is associated with multiple adverse outcomes for both mother and infant, including an increased risk of low birth weight, maternal mortality, perinatal mortality, and preterm birth, and is a recognized global problem [28]. This highlights the importance of ensuring an appropriate iron status during pregnancy and after delivery. Giving advice and ensuring that women in need of iron supplements receive it may be the most easily achieved measure to reduce MPRs in maternity care.

### Strengths and limitations

This study has several strengths, including both its size and the detail of data collected. The use of a standardized system for identifying MRPs, a standardized and systematic chart review at several points during hospitalization, and involvement of a panel of experts in MRP identification are additional important strengths of the present study. Moreover, health care personnel from Ethiopia with knowledge of the healthcare system, local language, culture, and previous research or practice experience performed the data collection.

A major limitation of this study is that our results depended on the accuracy of the chart recording by health professionals. Lack of recording/documentation occurs frequently due to lack of time, and the MRP identification panel agreed to consider it as a documentation problem rather than an MRP for the current study. This will result in an underestimation of the actual number of MRPs. Moreover, JUMC is a referral hospital with a larger proportion of women with pregnancy complications, and possibly with a higher need for medications. As such, the findings will probably not be representative of primary or secondary care services. Finally, as it was difficult to get the patient’s full pre-admission medication history, no formal medication reconciliation was performed and this could have underestimated the true prevalence of MRPs.

## Conclusions

This study confirms that MRPs are common among women in maternity and gynaecological wards. The most common MRPs were need for additional iron drug therapy, need for additional laboratory test, unnecessary drug therapy, and too low dose of medication. The most important factor associated with MRPs in pregnancy were parity, prior medication use, and chronic illness. Increased adherence to iron supplementation guidelines in inpatient maternity care is urgently needed. Future research should address whether interdisciplinary teams and clinical pharmacy services in the maternity and gynaecology ward can reduce the frequency and consequences of MRPs, and whether this leads to improvements in patient health outcomes.

## Supplementary Information

**Additional file 1:.** Identification, assessment, classification, and documentation of MRPs and recommendations by the panel of experts

**Additional file 2:.** Characteristics of the study population according to ward type at JUMC, Ethiopia, from February to June 2017

**Additional file 3:.** Medications used among hospitalized pregnant women at JUMC, Ethiopia, from February to June 2017

**Additional file 4:.** Discharge medications prescribed to hospitalized pregnant women at JUMC, Ethiopia, from February to June 2017

**Additional file 5:.** Types and examples of MRPs identified among hospitalized pregnant women at JUMC, Ethiopia, from February to June 2017

**Additional file 6:.** Medications involved in MRPs among hospitalized pregnant women at JUMC, Ethiopia, from February to June 2017

## Data Availability

The datasets used during the current study are available from the corresponding author upon reasonable request.

## Abbreviations

ANC: Antenatal care
ATC: Anatomical therapeutic chemical classification system
CI: Confidence interval
HEG: Hyperemesis gravidarum
JUMC: Jimma University medical centre
MMR: Maternal mortality ratio
MRP: Medication-related problem
OR: Odds ratio
WHO: World health Organization
