# Supplementary material for: Medication-related problems among hospitalized pregnant women in a tertiary teaching hospital in Ethiopia: a prospective observational study
Source: BMC Pregnancy Childbirth. 2020 Nov 26;20:737. doi: 10.1186/s12884-020-03433-6 (PMC7690074; doi:10.1186/s12884-020-03433-6)
Supplement: Supplementary file 1 — Additional file 1:. Identification, assessment, classification, and documentation of MRPs and recommendations by the panel of experts [file 12884_2020_3433_MOESM1_ESM.docx]

**Additional file 1. Identification,** **assessment, classification, and documentation of MRPs and recommendations by the panel of experts**

| **Medical Conditions & Medication Therapy Involved** | | | | | |
| --- | --- | --- | --- | --- | --- |
| **Medication-related needs** | **Categories of MRPs** | | **Subcategory or cause(s) of MRPs** | **Recommendation/intervention** | **Status of the recommendation** |
| **INDICATION** | - - - 1. **Needs additional drug therapy** | | - **Untreated medical condition** - **Preventive/ prophylactic** - **Synergistic/ potentiating** - **Others** | - **Initiate new drug therapy** - **Other** | - **Accepted,** - **Partially accepted** - **Rejected** |
|  | - - - 1. **Unnecessary drug therapy** | | - **No medical indication** - **Duplicate therapy** - **Non-drug therapy indicated** - **Treating avoidable ADR** - **Addictive/ recreational** - **Others** | - **Discontinue drug therapy** - **Initiate non drug therapy** - **Patient counseling** - **Other** | - **Accepted,** - **Partially accepted** - **Rejected** |
| **EFFECTIVENESS** | - - - 1. **Dose too low** | | - **Wrong dose** - **Frequency inappropriate** - **Duration too short** - **Drug interaction** - **Others** | - **Increase dose** - **Increase frequency** - **Avoid interacting drugs** - **Prolong duration** - **Other** | - **Accepted,** - **Partially accepted** - **Rejected** |
|  | - - - 1. **Ineffective drug** | | - **More effective drug available** - **Condition refractory to drug** - **Dosage form inappropriate** - **Not effective for condition** - **Others** | - **Switch to more effective agent** - **Institute a monitoring plan or recommend lab/other test** - **Switch to preferred dosage form or route** - **Other** | - **Accepted,** - **Partially accepted** - **Rejected** |
| **SAFETY** | - - - 1. **Dose too high** | | - **Wrong dose** - **Frequency inappropriate** - **Duration too long** - **Incorrect administration** - **Drug interaction** - **Others** | - **Decrease dose** - **Change duration** - **Change frequency** - **Other** | - **Accepted,** - **Partially accepted** - **Rejected** |
|  | - - - 1. **Adverse drug reaction** | | - **Undesirable effect** - **Unsafe drug for patient** - **Drug interaction** - **Dosage administered or changed too fast** - **Allergic reaction** - **Contraindications present** - **Others** | - **Institute a monitoring plan or recommend lab/other test** - **Switch to safer alternative** - **Dose titration** - **Other** | - **Accepted,** - **Partially accepted** - **Rejected** |
| **ADHERENCE** | - - - 1. **Compliance** | | - **Directions not understood** - **Patient prefers not to take** - **Patient forgets to take** - **Drug product too expensive** - **Patient cannot swallow/ administer** - **Drug product not available** - **Patient felt better or worse** - **Regimen complexity** - **Fear of adverse events** - **Patient not aware of medication changes** - **Disbelief in drug effectiveness** - **Others** | - **Provide patient-specific instructions** - **Change administration time, route or dosage form** - **Switch to generic alternative** - **Switch to cheaper alternative** - **Switch to safer alternative** - **Provide adherence aid or educate** - **Others** | - **Accepted,** - **Partially accepted** - **Rejected** |
| **OTHER CATEGORIES** | - - - 1. **Other Categories** | **Need for additional laboratory test** | - **laboratory parameters are required to determine if the patient needs treatment** | - **Order laboratory tests** | - **Accepted,** - **Partially accepted** - **Rejected** |
|  |  | **Incomplete drug order** | **Patient is on a medication where a particular detail of the order has been omitted** | - **Correct the errors in the medication order** | - **Accepted,** - **Partially accepted** - **Rejected** |
