# Supplementary material for: Medication-related problems among hospitalized pregnant women in a tertiary teaching hospital in Ethiopia: a prospective observational study
Source: BMC Pregnancy Childbirth. 2020 Nov 26;20:737. doi: 10.1186/s12884-020-03433-6 (PMC7690074; doi:10.1186/s12884-020-03433-6)
Supplement: Supplementary file 2 — Additional file 2:. Characteristics of the study population according to ward type at JUMC, Ethiopia, from February to June 2017 [file 12884_2020_3433_MOESM2_ESM.docx]

**Additional file 2.** Characteristics of the study population according to ward type at JUMC, Ethiopia, from February to June 2017

| Characteristic | Total | Maternity ward | Gynecology ward |
| --- | --- | --- | --- |
| Number of women, n | 1117 | 992 | 125 |
| Age (years), median (range) | 25 (18-45) | 26 (18-45) | 25 (18-45) |
| Length of stay (days), median (range) | 3 (0.2-60) | 3 (0.2-60) | 3 (1-27) |
| Number of patients who used medicines during pregnancy, n (%)* | 952 (85.2) | 901 (90.8) | 51 (40.8) |
| Number of medicines during pregnancy, median (range) | 1 (0-6) | 1 (0-6) | 0 (0-5) |
| Number of patients who used medicinal plants during pregnancy, n (%) | 319 (28.6) | 297 (26.6) | 22 (2.0) |
| Number of medicinal plants used per woman during pregnancy, median (range)** | 2 (1-8) | 2 (1-8) | 3 (1-5) |
| *During admission* | | | |
| Number of patients who used medicines during admission, n (%)*** | 1101 (98.6) | 985 (99.3) | 116 (92.8) |
| Number of medicines during admission, median (range)******** | 3 (0-24) | 4 (0-24) | 3 (0-10) |
| Number of chart reviews performed, median (range) | 2 (1-3) | 2 (1-3) | 2 (1-3) |
| Number of MRPs identified, n (%)***** | 394 (100.0) | 345 (87.6) | 49 (12.4) |
| Number of patients with ≥1 MRP, n (%) | 323 (28.9) | 276 (24.7) | 47 (4.2) |
| Median number of MRPs identified (range) | 1 (1-4) | 1 (1-4) | 1 (1-2) |
| Level 2 MRPs, n (%) | 278 (70.6) | 237(68.7) | 41(83.7) |

Abbreviations: MRP, medication-related problem;

* Considering medicines used before admission to the hospital

** Considering those who used medicinal plants

*** Considering medicines used after admission to the hospital

**** Number of regular medications recorded as an inpatient was recorded at the point of initial review and updated during subsequent chart reviews if additional regular medicines were registered and finally crosschecked on or after discharge.

***** Considering those who encounter ≥1 MRP
