# Supplementary material for: Medication-related problems among hospitalized pregnant women in a tertiary teaching hospital in Ethiopia: a prospective observational study
Source: BMC Pregnancy Childbirth. 2020 Nov 26;20:737. doi: 10.1186/s12884-020-03433-6 (PMC7690074; doi:10.1186/s12884-020-03433-6)
Supplement: Supplementary file 3 — Additional file 3:. Medications used among hospitalized pregnant women at JUMC, Ethiopia, from February to June 2017 [file 12884_2020_3433_MOESM3_ESM.docx]

**Additional file 3.** Medications used among hospitalized pregnant women at JUMC,

Ethiopia, from February to June 2017

| Medications used (N=1117) | Frequency | Percentage* |
| --- | --- | --- |
| Pitocin | 711 | 63.7 |
| Normal Saline (NS) iv solution | 434 | 38.9 |
| Ceftriaxone | 402 | 36.0 |
| Ringer's lactate (RL) iv solution | 366 | 32.8 |
| Tramadol | 364 | 32.6 |
| Dextrose in normal saline (DNS) iv solution | 332 | 28.8 |
| Bupivacaine | 254 | 22.7 |
| Ferrous sulphate | 222 | 19.9 |
| Magnesium sulfate | 181 | 16.2 |
| Metronidazole | 170 | 15.2 |
| Metoclopramide | 154 | 13.8 |
| Pethidine | 141 | 12.6 |
| Dexamethasone | 130 | 11.6 |
| Suxamethonium chloride | 103 | 9.2 |
| Ketamine | 97 | 8.7 |
| Cephalexin | 82 | 7.3 |
| Methyl dopa | 83 | 7.4 |
| Paracetamol | 77 | 6.9 |
| Atropine | 74 | 6.6 |
| Misoprostol | 56 | 5.0 |
| Ergometrine | 55 | 4.9 |
| Blood (1 unit - 4 units) | 57 | 5.1 |
| Diazepam | 41 | 3.7 |
| Mifepristone | 37 | 3.3 |
| Hydralazine | 35 | 3.1 |
| Dextrose 40% | 34 | 3.0 |
| Doxycycline | 31 | 2.8 |
| Propofol | 30 | 2.7 |
| Diclofenac | 29 | 2.6 |
| Nifedipine | 28 | 2.5 |
| Erythromycin | 25 | 2.2 |
| Lidocaine | 23 | 2.1 |
| Ibuprofen | 20 | 1.8 |
| Thiopentone | 19 | 1.7 |
| Furosemide | 17 | 1.5 |
| Anti-D | 16 | 1.4 |
| Vitamin B complex | 13 | 1.2 |
| Cimetidine | 13 | 1.2 |
| Amoxicillin | 8 | 0.7 |
| Vancomycin | 5 | 0.5 |
| Vancuronium | 5 | 0.5 |
| Neostigmine | 5 | 0.4 |
| Folic acid | 5 | 0.4 |
| Adrenaline | 4 | 0.4 |
| Ciprofloxacin | 3 | 0.3 |
| Propylthiouracil | 3 | 0.3 |
| Ceftazidime | 3 | 0.3 |
| Propranolol | 3 | 0.3 |
| Artesunate | 3 | 0.3 |
| Highly active antiretroviral therapy (HAART) | 3 | 0.3 |
| Norfloxacin | 3 | 0.3 |
| Omeprazole | 2 | 0.2 |
| Ferrous fumarate | 2 | 0.2 |
| Promethazine | 2 | 0.2 |
| Azithromycin | 2 | 0.2 |
| Amlodipine | 2 | 0.2 |
| Multivitamin | 2 | 0.2 |
| Ampicillin | 2 | 0.2 |
| Plumppy nut | 2 | 0.2 |
| Hydrocortisol | 2 | 0.2 |
| Potassium chloride | 2 | 0.2 |
| Chlorpromazine | 2 | 0.2 |
| Others ** | 17 | 1.5 |

*Percentage is calculated taking the final study population ready for analysis, N=1117

*Percentage may exceed 100% due to multiple responses

**Others: Ferrous gluconate, Salbutamol, Oxygen, Lugol's solution, Vitamin K, Phenytoin, Albendazole, Mebendazole, Calcium

gluconate, Clindamycin, Gentamycin, Chloroamphonicol, Chloroquine, Digoxin, Gallamine triethiodide, Rocuronium bromide, Haloperidol, (each with one frequency)
