# Supplementary material for: Medication-related problems among hospitalized pregnant women in a tertiary teaching hospital in Ethiopia: a prospective observational study
Source: BMC Pregnancy Childbirth. 2020 Nov 26;20:737. doi: 10.1186/s12884-020-03433-6 (PMC7690074; doi:10.1186/s12884-020-03433-6)
Supplement: Supplementary file 4 — Additional file 4:. Discharge medications prescribed to hospitalized pregnant women at JUMC, Ethiopia, from February to June 2017 [file 12884_2020_3433_MOESM4_ESM.docx]

**Additional file 4.** Discharge medications prescribed to hospitalized pregnant women at JUMC, Ethiopia, from February to June 2017

| Discharge Medications Types (N= 555) | Frequency | Percentage* |
| --- | --- | --- |
| Ferrous sulphate | 302 | 54.4 |
| Cephalexin | 168 | 30.4 |
| Metronidazole | 139 | 25.0 |
| Methyl dopa | 82 | 14.8 |
| Doxycycline | 57 | 10.3 |
| Tramadol | 49 | 8.8 |
| Implanon | 39 | 7.0 |
| Ibuprofen | 17 | 3.1 |
| Metoclopramide | 14 | 2.5 |
| Paracetamol | 13 | 2.3 |
| Folic acid | 8 | 1.4 |
| Erythromycin | 7 | 1.3 |
| Amoxicillin | 5 | 0.9 |
| Nifedipine | 5 | 0.9 |
| Furosemide | 5 | 0.9 |
| Ferrous fumarate | 5 | 0.9 |
| Nevirapine | 5 | 0.9 |
| Highly active antiretroviral therapy (HAART)** | 4 | 0.7 |
| Anti-D | 4 | 0.7 |
| Diclofenac | 3 | 0.5 |
| Depo provera | 3 | 0.5 |
| Amlodipine | 3 | 0.5 |
| Propranolol | 3 | 0.5 |
| Ciprofloxacin | 2 | 0.4 |
| Multivitamin | 2 | 0.4 |
| Propylthiouracil | 2 | 0.4 |
| Pyridoxine | 2 | 0.4 |
| Others *** | 12 | 2.4 |

*Percentage may exceed 100% due to multiple responses

**Highly active antiretroviral therapy (HAART) is the use of multiple drugs that act on different viral targets.

***Others: Fefol spansule capsules, Vitamin B complex, Omeprazole, Ferrous gluconate, Intrauterine contraceptive device (IUCD), Promethazine, Pantoprazole, Salbutamol, Insulin, Bisacodyl, Ampicillin, and Digoxin, each with one frequency
