# Supplementary material for: Medication-related problems among hospitalized pregnant women in a tertiary teaching hospital in Ethiopia: a prospective observational study
Source: BMC Pregnancy Childbirth. 2020 Nov 26;20:737. doi: 10.1186/s12884-020-03433-6 (PMC7690074; doi:10.1186/s12884-020-03433-6)
Supplement: Supplementary file 5 — Additional file 5:. Types and examples of MRPs identified among hospitalized pregnant women at JUMC, Ethiopia, from February to June 2017 [file 12884_2020_3433_MOESM5_ESM.docx]

**Additional file 5.** Types and examples of MRPs identified among hospitalized pregnant women at JUMC, from Ethiopia, February to June 2017

| Type (cause) of MRP | n (%) | Example of MRPs |
| --- | --- | --- |
| Untreated condition | 199 (61.6%) | Patient had post cesarean section hematocrit value of 25%, however, she was not taking iron treatment |
| Need for an additional laboratory test | 41 (12.7%) | Patient hematocrit value is not recorded to recommend or not iron supplementation or treatment |
| Preventive or prophylactic | 37 (11.5%) | Patient is at risk of infection due to retained placenta, however prophylactic antibiotic treatment was not indicated in the charts |
| No medical condition | 32 (9.9%) | The patient was given oral amoxicillin and metronidazole. However, patient had neither risk of infection nor indication for the antibiotics |
| Medication interaction | 24 (7.4%) | Patient is given oral iron sulphate and doxycycline concurrently that will lead to medication interaction and less medication absorption. Administer doxycycline 2 hours before or 4 hours after iron sulphate |
| More effective drug available | 10 (3.1%) | Patient with gestational hypertension was put on furosemide. According to the guideline the more effective methyldopa should have been used (first drug of choice). |
| Drug product too expensive | 7 (2.2%) | Although anti-D immunoglobulin is available in the hospital, the patient could not afford it and was not injected |
| Dosage too low | 6 (1.9%) | Patient with uncontrolled diabetes mellitus that needs tight glucose control was on insulin 0.5 IU/kg/day treatment which is too low dose; The dose should be adjusted to 0.75 IU/kg/day. |
| Duplicate therapy | 6 (1.9%) | Patient is on pethidine followed by tramadol to relieve pain. Moreover, the patient is also on diclofenac 75 mg iv PRN. This therapeutic duplication may lead to an unintended overdose, as well as potential adverse drug reaction |
| Duration inappropriate (long duration) | 6 (1.9%) | Patient with hematocrit value of 32.8% was prescribed iron sulphate 325 mg Po/TID for 6 months. 3 months duration was considered appropriate for this patient. |
| Duration inappropriate (short duration) | 6 (1.9%) | Patient received ceftriaxone 1g iv BID for one day only. Treatment should have been continued for 5-7 days or should have been changed to oral medications. |
| Dosage too high | 5 (1.5%) | Patient received pethidine 50 mg im TID, while 50 mg as needed was appropriate (scheduled basis treatment can even lead to addiction) |
| Drug product not available | 3 (0.9%) | Patient is hepatitis B surface antigen (HBsAg) test positive, however, the treatment is not available in the country. |
| Incomplete drug order | 3 (0.9%) | Patient is receiving first-line agent methyl dopa 250 mg for severe preeclampsia treatment but duration was not specified |
| Directions not understood | 2 (0.6%) | Patient is prescribed cephalexin and erythromycin, however, both medications were not bought as the patient did not understand the health professional’s directions |
| Dosage form/ route inappropriate | 2 (0.6%) | Patient is in comma; however, instead of the parenteral route she was given oral methyldopa and haloperidol |
| Frequency inappropriate (low dose) | 2 (0.6%) | Patient is on oral cephalexin 500 mg once daily to prevent risk of infection. This frequency is too low and should be increased to twice per day. |
| Contraindication present | 1 (0.3%) | SPE woman who was also an AKI patient; she was on magnesium sulphate (for convulsion prevention) which can lead to magnesium intoxication so as an alternative diazepam indicated |
| Frequency inappropriate (Safety) | 1 (0.3%) | Patient did not receive one dose magnesium sulphate because the nurse forgot to administer. Patient was suffering from severe preeclampsia |
| Unsafe medication | 1 (0.3%) | Patient received furosemide and gentamicin concurrently. Either increases toxicity (ototoxicity and nephrotoxicity) of the other by pharmacodynamic synergism; alternative drug chlorothiazide was used in place of furosemide |

*Percentage may exceed 100% due to more than one MRP per patient

*Percentage is calculated taking those with ≥ MRP as denominator

Abbreviations: MRP, medication-related problem; BID, twice a day; TID three times a day; IM, intramuscular; IV, intravenous; SPE, Severe pre-eclampsia; AKI, Acute kidney injury; PRN, as needed
