# Supplementary material for: Medication-related problems among hospitalized pregnant women in a tertiary teaching hospital in Ethiopia: a prospective observational study
Source: BMC Pregnancy Childbirth. 2020 Nov 26;20:737. doi: 10.1186/s12884-020-03433-6 (PMC7690074; doi:10.1186/s12884-020-03433-6)
Supplement: Supplementary file 6 — Additional file 6:. Medications involved in MRPs among hospitalized pregnant women at JUMC, Ethiopia, from February to June 2017 [file 12884_2020_3433_MOESM6_ESM.docx]

**Additional file 6.** Medications involved in MRPs among hospitalized pregnant women at JUMC,

Ethiopia, from February to June 2017

| Medications involved in MRP | Frequency | Percentage* |
| --- | --- | --- |
| Ferrous sulphate | 139 | 35.3 |
| Methyldopa | 54 | 13.7 |
| Cephalexin | 37 | 9.4 |
| Metronidazole | 37 | 9.4 |
| Doxycycline | 22 | 5.6 |
| Ceftriaxone | 13 | 3.3 |
| Anti-D | 10 | 2.5 |
| Tramadol | 9 | 2.3 |
| Amoxicillin | 6 | 1.5 |
| Furosemide | 5 | 1.3 |
| Magnesium sulfate | 3 | 0.8 |
| Erythromycin | 3 | 0.8 |
| Metoclopramide | 3 | 0.8 |
| Ciprofloxacin | 2 | 0.5 |
| Gentamycin | 2 | 0.5 |
| Diclofenac | 2 | 0.5 |
| Paracetamol | 2 | 0.5 |
| Diazepam | 2 | 0.5 |
| HBV vaccine | 2 | 0.5 |
| Nifedipine | 2 | 0.5 |
| Amlodipine | 2 | 0.5 |
| Need for an additional laboratory test | 41 | 10.4 |
| Others ** | 18 | 4.6 |

*Percentage is calculated taking total MRPs (N=394) as denominator;

*Percentage may exceed 100% due to multiple responses

**Aspirin, Azithromycin, Calcium gluconate, Chemotherapy/Surgery/Radiation, Chloroquine, Cimetidine, Clindamycin, Ferrous fumarate, Haloperidol, Ibuprofen, Insulin, Norfloxacin, Pethidine, Propylthiouracil, Ringer lactate, Salbutamol, Vancomycin, Ampicillin
